# Ultrasound in chronic liver disease

**DOI:** 10.1007/s13244-014-0336-2

**Published:** 2014-05-24

**Authors:** J. F. Gerstenmaier, R. N. Gibson

**Affiliations:** Department of Radiology, (RNG also University of Melbourne) The Royal Melbourne Hospital, Grattan Street, Parkville, VIC 3050 Australia

**Keywords:** Fibrosis, Cirrhosis, Fatty liver disease, Elastography, Ultrasound, Contrast-enhanced ultrasound, Doppler, Hepatocellular carcinoma, Portal venous hypertension

## Abstract

**Background:**

With the high prevalence of diffuse liver disease there is a strong clinical need for noninvasive detection and grading of fibrosis and steatosis as well as detection of complications.

**Methods:**

B-mode ultrasound supplemented by portal system Doppler and contrast-enhanced ultrasound are the principal techniques in the assessment of liver parenchyma and portal venous hypertension and in hepatocellular carcinoma surveillance.

**Results:**

Fibrosis can be detected and staged with reasonable accuracy using Transient Elastography and Acoustic Radiation Force Imaging. Newer elastography techniques are emerging that are undergoing validation and may further improve accuracy. Ultrasound grading of hepatic steatosis currently is predominantly qualitative.

**Conclusion:**

A summary of methods including B-mode, Doppler, contrast-enhanced ultrasound and various elastography techniques, and their current performance in assessing the liver, is provided.

***Teaching Points*:**

*• Diffuse liver disease is becoming more prevalent and there is a strong clinical need for noninvasive detection.*

*• Portal hypertension can be best diagnosed by demonstrating portosystemic collateral venous flow.*

*• B-mode US is the principal US technique supplemented by portal system Doppler.*

*• B-mode US is relied upon in HCC surveillance, and CEUS is useful in the evaluation of possible HCC.*

*• Fibrosis can be detected and staged with reasonable accuracy using TE and ARFI.*

*• US detection of steatosis is currently reasonably accurate but grading of severity is of limited accuracy.*

## Introduction

Ultrasound (US) has a major role in the diagnosis and management of chronic liver diseases by providing diagnostic and prognostic information as well as detecting complications such as HCC and portal hypertension. While conventional ultrasound is valuable in the assessment of liver parenchyma and detection of liver lesions, a range of other US techniques has been developed that increases its potential value. Noninvasive methods of measurements in chronic liver disease are rapidly changing in performance capabilities and availability. These include laboratory tests and imaging studies. An area of intense recent interest has been elastography because of its ability to provide noninvasive information about the stage of liver fibrosis.

The purpose of this review is to summarise the range of US techniques now available and to provide some perspective on their current and potential future value, with a particular focus on elastography, one of the techniques now in mainstream use.

## The clinical challenge—fibrosis and steatosis detection and grading

Hepatic fibrosis is a response to chronic liver injury and a process that tends to progress to cirrhosis and end-stage liver disease. While alcohol and infection with hepatitis B virus (HBV) and hepatitis C virus (HCV) are still the leading causes worldwide, the increasing prevalence of metabolic syndrome and obesity has resulted in an increasing incidence of cirrhosis secondary to non-alcoholic fatty liver disease (NAFLD). The prevalence of NAFLD is higher than previously estimated [1]. If the incidences of obesity and diabetes continue to rise at the current rate, the prevalence of NAFLD in the US is expected to exceed 50 % in 2030, reaching epidemic status [2]. Non-alcoholic steatohepatitis (NASH), first described in 1980 [3], is a severe and progressive form of NAFLD and is now recognised as a major cause of cirrhosis.

Most histological staging systems for fibrosis and cirrhosis provide five stages, e.g. the METAVIR [4]: stage 0 (F0) = normal connective tissue; stage 1 (F1) = fibrous portal expansion; stage 2 (F2) = periportal or scanty porto-portal septa; stage 3 (F3) = fibrous septa with architectural distortion; stage 4 (F4) = cirrhosis. Stages 2 and 3 are considered significant and severe fibrosis respectively. Staging liver fibrosis is important for several reasons: in chronic viral disease, identification of the severity of the liver damage is necessary in order to allow a timely treatment start to avoid progression to cirrhosis when fibrosis stage 2 or beyond is present; the monitoring for progression or regression of liver fibrosis during treatment; and the commencement of monitoring for complications (HCC, PHT) in fibrosis stage 3 or cirrhosis.

Macrovesicular steatosis of the liver can be graded S0–S3 based on percent of hepatocytes in the biopsy involved (S0 is none; S1 is up to 33 %; S2 is 33–66 %; S3 is >66 %) [5]. The grade of steatosis is one parameter in the staging for NASH, e.g. used in the the NASH CRN scoring system [6]. Very similar to the grading system proposed by Brunt et al. is the NASH revised criteria used for histological scoring by Dixon with grades 0–4 assigned to <5 %, 5–25 % 25–50 %, 50–75 % and >75 % respectively [7].

With emerging treatments for hepatic fibrosis and NAFLD there is growing demand for accurate diagnosis, prognosis and monitoring of the disease. Traditionally, liver biopsy has been considered the gold standard in fibrosis assessment [8, 9]. Liver biopsy has a number of disadvantages. As an invasive test, it has a complication rate of approximately 1 % [10–12]. Liver biopsy has been shown to have a high rate of sampling error in patients with diffuse parenchymal liver diseases. A typical specimen volume taken at core biopsy represents only 1/50,000 of liver volume [13]; however fibrosis is heterogeneously distributed in the liver. As an example, in a series of 124 HCV patients, samples taken from the right and left hepatic lobes differed in histological grading and staging. As a result of sampling error, underdiagnosis of cirrhosis occurred in 14.5 % of the patients [14].

## Ultrasound modalities

### B-mode ultrasound

#### Fatty liver disease

**Fig. 1:**
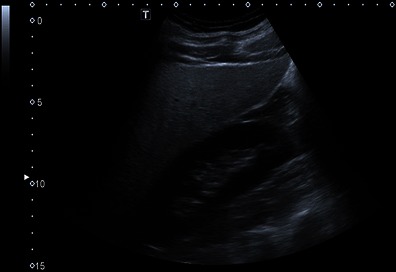
B-mode ultrasound of liver and right kidney. Diffuse fatty infiltration. The liver is of markedly increased echo intensity relative to the renal cortex, indicative of severe steatosis

At conventional B-mode ultrasound, diffuse fatty infiltration results in increased echogenicity of the liver when compared to other organs such as the renal cortex (Fig. 1). Features include increased echogenicity of the liver parenchyma, poor or non-visualisation of the diaphragm, intrahepatic vessels and posterior part of the right hepatic lobe. Qualitative grading is conveniently made as mild, moderate or severe, or grade 0–3 with 0 being normal. Grade 1 (mild) is represented by a diffuse slight increase in fine echoes in the hepatic parenchyma with normal visualisation of the diaphragm and intrahepatic vessel borders. Grade 2 (moderate) is represented by a moderate diffuse increase in fine echoes with slightly impaired visualisation of the intrahepatic vessels and diaphragm. Grade 3 (marked) is represented by a marked increase in fine echoes with poor or no visualisation of the intrahepatic vessel borders, diaphragm and posterior portion of the right lobe of the liver [15]. Focal fatty sparing may be seen in grades 2 and 3. A de facto validation of qualitative grading has occurred in a meta-analysis that included data from studies comparing US with histopathological findings. Ultrasound was found to have acceptable diagnostic accuracy for detecting moderate or severe hepatic steatosis in adults, with pooled sensitivities ranging from 0.857 to 0.991 and pooled specificities ranging from 0.852 to 0.919 [16]. Cutoff values for grading steatosis varied across studies included in the meta-analysis but were not less than 25 % for moderate to severe hepatic steatosis. The sensitivities and specificities for mild (less than 5 %) steatosis were 0.733 and 0.844 respectively.

Data in the adolescent population are scarce. In a small series of 34 overweight Egyptian children with liver biopsy, qualitative ultrasound scores of 0 or 1 were found to exclude histological NAFLD [17]. In an earlier prospective study of 104 adolescents, qualitative scores 0–3 for the presence of fatty infiltration at ultrasound were compared with MR spectroscopy results. While negative US results excluded the presence of severe steatosis with acceptable accuracy, positive US results in severely obese adolescents could not be used to accurately predict the presence and severity of hepatic steatosis [18].

**Fig. 2:**
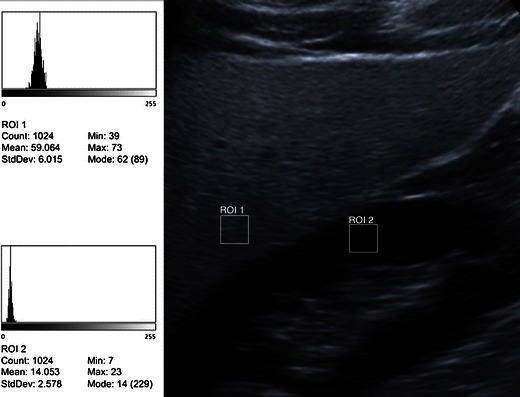
Quantification of echo intensity. Principle of the sonographic hepatorenal index. ROIs are placed over the liver parenchyma and renal cortex at the same depth. Mean echo intensities are determined on a standard 8-bit US image, e.g. in this example with freeware ImageJ, National Institutes of Health, Bethesda, MD, USA). SHRI is calculated by dividing the mean liver brightness by the mean renal cortex brightness. In this example 59/14 = 4.21, i.e. severely fatty [20–23]

Due to substantial inter- and intraobserver variability [19] and the reduced sensitivity in low levels of steatosis [16, 20], it has been suggested that the effectiveness of steatosis detection can be increased by quantification of liver brightness. The sonographic hepatorenal index (SHRI) is based on comparison between liver and kidney brightness. An image including both liver and kidney is required, typically showing segment 6 of the liver and the upper pole of the right kidney. Regions of interest (ROI) of an appropriate size (>400 pixels) are selected in the liver parenchyma, excluding vessels, and renal cortex at the same field depth. The mean brightness of each ROI is determined using numerical values assigned to grey-scale pixels. Some ultrasound systems allow the placement of ROIs directly on the screen. Alternatively, a suitable image can be exported and ROIs placed using proprietary software or public domain programmes such as ImageJ (National Institutes of Health, Bethesda, MD, USA). The SHRI is the mean liver brightness divided by the mean renal cortex brightness (Fig. 2). Significant correlation between histological steatosis and the SHRI has been found in several studies. In addition, point estimates of SHRI for the prediction of steatosis grades less than moderate or severe appear to be superior to those of qualitative grading methods. In a series of 101 patients who underwent liver biopsy, a strong correlation between the SHRI and percentage of fat was shown (Spearman’s coefficient = 0.71, *P* < 0.001) [21]. In this study, liver biopsy was performed for a variety of reasons including elevated liver function tests, HCV, orthotopic liver transplant and HBV. A SHRI cutoff of 1.28 had a sensitivity of 1 and a specificity of 0.54 for the diagnosis of steatosis greater than 5 %. The authors reported that more than 1/3 of biopsies could have been avoided in their series of 101 patients if the SHRI method had been used prospectively. In another study of 42 NAFLD patients and 40 healthy controls, the SHRI cutoff for predicting steatosis was 1.24 with a sensitivity of 0.93 and a specificity of 0.93 [22]. In a series of 111 consecutive patients undergoing liver biopsy for a variety of indications including HBV, HBC and abnormal liver enzymes, the SHRIs were determined retrospectively. An SHRI cutoff point of 1.49 had a sensitivity of 1 and specificity of 0.91 for the prediction of steatosis >5 % [20]. In a recent study involving patients attending general medical centres, SHRI as determined on a standard workstation without additional software showed strong correlation (Spearman’s coefficient = 0.89, *P* < 0.001) with 3T MR proton spectroscopy as a reference to determine the degree of steatosis [23]. The authors found that SHRI cutoff points of 1.21, 1.28 and 2.15 yielded 100 % sensitivity for the diagnoses of steatosis greater than 5 %, 25 % and 50 %, respectively, with a specificity greater than 70 %. The degrees of steatosis referred to in this study correspond to those in the Dixon steatosis grading system [7]. It appears that amongst the limited number of studies to date, no uniformly agreed SHRI threshold for cutoffs of degrees of steatosis have been found.

Regarding reproducibility, repeat measurements of the SHRI in a sample of hospital workers were made several days apart by one sonographer. An r value of 0.77 for correlation between the two measurements was achieved. A kappa value of 0.86 when applying a cutoff point of 1.49 for the diagnosis of steatosis indicates very good intraobserver agreement [20]. Interobserver agreement for the SHRI has not been assessed.

In summary, the SHRI appears to be an appealing technique for diagnosis and quantification of hepatic steatosis. It can be performed without new investment, but a standardised technique and interobserver agreement still need to be determined.

**Fig. 3:**
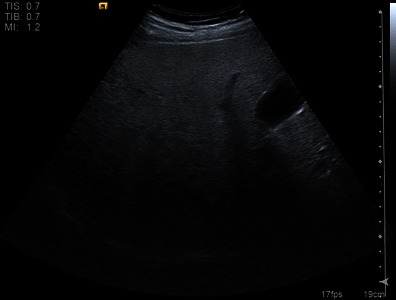
Tissue harmonic compound sonography. Signal drop in the far field (fade-out sign) indicated severe diffuse fatty infiltration

Lee et al. describe a “dark band at the posterior deep portion of the liver” [24] on tissue harmonic compound sonography (Fig. 3). This becomes especially conspicuous when the fundamental mode of compound sonography is converted to the tissue harmonic compound sonography mode for evaluation of focal hepatic lesions. We have also observed this phenomenon. Compound sonography has the advantage of better sharpness and contrast, and an improved signal-to-noise ratio. Especially when combined with tissue harmonic imaging, noise is kept to a minimum and some artefacts such as reverberation are removed, ideal for investigating a focal lesion [25–27]. As a trade-off, posterior shadowing may become more conspicuous. An explanation of the dark area could be an abrupt drop in the harmonic signal in deeper portions due to the effect of fatty infiltration on acoustic penetration. Termed the “fade-out sign”, this phenomenon was investigated for its value in diagnosing fatty liver disease [24]. At this time the fade-out signal appears to be an observed qualitative feature of hepatic steatosis at harmonic US imaging.

#### Echotexture analysis

A feature of ultrasound is the presence of speckle noise. Within an image, speckle is an intensity pattern formed by the interference of many scatters and not a direct representation of the underlying structure. However, the local brightness of the speckle reflects the corresponding echogenicity of the underlying scatterers, in the case of liver ultrasound likely the hepatic lobules.

**Fig. 4:**
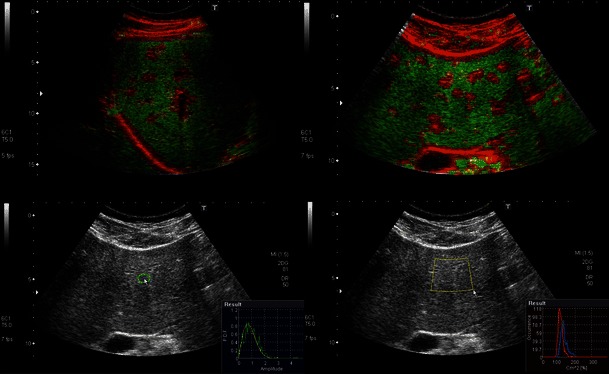
Speckle pattern analysis with acoustic structure quantification (ASQ). *Upper images*: Homogeneous normal liver parenchyma is displayed as green whereas portal tracts and other structures with different and heterogeneous structure are highlighted in red. *Lower images*: Example of quantitative tools showing the probability density function (*green curve*) and theoretical speckle generated by Rayleigh distribution (*red*)

The speckle pattern changes with both steatosis and fibrosis. Microarchitectural changes may be imperceptible to the naked eye on a conventional B-mode image. Acoustic structure quantification (ASQ) is one method of quantifying the statistical deviation of ultrasound signals that occurs in diffuse pathological processes [28]. In the evaluation of fibrosis and cirrhosis, ASQ as yet has not been shown to perform as well as TE [29]. ASQ technology has been proposed for the quantification of steatosis [30]. Figure 4 shows an example of how ASQ is displayed.

#### Fibrosis and cirrhosis

**Fig. 5:**
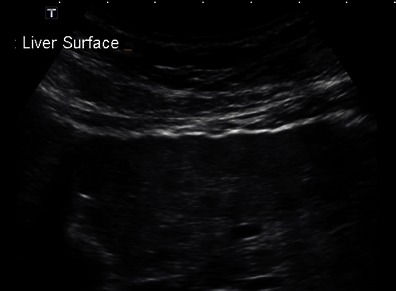
Example of surface nodularity

Liver parenchymal texture is a characteristic that is somewhat subjective and has low sensitivity for the detection of cirrhosis. A recent retrospective study on the accuracy of conventional US in the staging of fibrosis found that routine US is not an accurate predictor for either early or significant fibrosis in chronic viral hepatitis [31]. However in a series of 103 patients with chronic liver disease it has been shown that liver parenchymal texture (graded as fine echotexture, mildly coarse, coarse and highly coarse) has a statistically significant correlation (rs = 0.8853) with the degree of fibrosis [32]. When combined with two more features (liver surface nodularity and liver edge), correlation with the degree of fibrosis increased to rs = 0.9524. When compared to echotexture, liver surface nodularity (Fig. 5) has better accuracy for the presence of cirrhosis [33–35] reaching both a sensitivity and specificity of 0.88. In order to provide a fluid-tissue interface, ascites needs to be present for optimal evaluation. Once ascites is present, cirrhosis is generally more advanced and less of a diagnostic challenge.

**Fig. 6:**
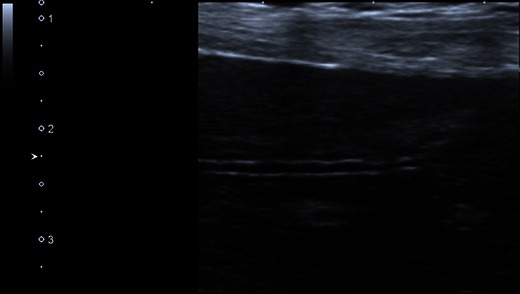
Hepatic vein wall morphology. Against contrast of anechoic blood within the hepatic vein, the wall appears wavy, concurring with the known diagnosis of cirrhosis. In the absence of ascites, surface nodularity may not be as conspicuous as in Fig. 5

A different approach is the use of the hepatic vein lumen as an internal fluid-tissue interface when ascites is absent (Fig. 6). Assuming that internal nodularity in cirrhosis would cause architectural distortion, the hepatic vein morphology would also be altered. In a prospective pilot study comprising 38 patients with cirrhosis and 50 patients without liver disease, the following features were evaluated: hepatic vein straightness, uniformity of hepatic vein echogenicity and visualisation of a 1-cm segment of hepatic vein [36]. Hepatic vein straightness, stratified into three categories (straight, slightly wavy and very wavy) yielded the highest sensitivity and specificity of 0.97 and 0.91 respectively using real-time compound imaging (RTCI) with a 5–2 MHz transducer. Uniformity of hepatic vein wall echogenicity was the next useful feature with a sensitivity and specificity of 0.88 and 0.86 respectively, similar to earlier studies on superficial surface nodularity. With all three features combined, specificity for cirrhosis reached 0.98 using RTCI; however sensitivity reduced to 0.65. In this pilot study, the assessment of the hepatic vein morphology has been shown to be a good indicator of cirrhosis with favourable inter- and intraobserver error. While validation with more rigorous clinicopathological correlation and larger patient numbers is required, the three hepatic vein morphology characteristics can easily be evaluated in clinical practice. The authors now recommend that examination of the hepatic vein wall should preferably be performed in segment 5 or 6. A peripheral tributary should be selected perpendicular to the ultrasound beam in order to achieve a good specular reflection. The peripheral tributary should measure approximately 3 mm in diameter and at least 15 mm in length; choosing a peripheral tributary also allows the use of higher resolution scanning [37].

While hepatic vein wall morphology appears to be a feature with excellent accuracy for the diagnosis of cirrhosis in the pilot study [36], a recent study was not able to demonstrate this high sensitivity and reported liver surface nodularity to be more sensitive [38]. It is likely that these discrepant findings are technique related. When applying a meticulous technique as detailed in Gibson et al. [37], the authors’ experience is that diagnostic confidence is higher with hepatic vein morphology than with surface nodularity.

Portal vein diameter is known to increase following a meal, and the effect can be as much as 50 % [39]. In a small series, this effect has been shown to be present in normal subjects and chronic hepatitis patients alike, but in liver cirrhosis mean calibre, mean flow velocity and mean flow volume remained largely unchanged after a meal [40]. Absolute portal vein calibre has been considered a sign of portal venous hypertension with cutoff values of 13–15 mm [41–43], however with poor sensitivities of 0.13–0.4. The lack of sensitivity is likely due to the presence of collateral pathways that decompress the system. An angiography-based study concurred with the finding that portal vein diameter did not increase along with the portohepatic gradient in portal venous hypertension [44]. In our experience, the portal vein diameter may even appear small in the setting of hepatofugal flow. In our practice little significance is attached to this parameter as it has low positive predictive value for portal hypertension and a small percent of normals have portal vein diameters >13 mm. Portal vein diameter should therefore be interpreted with caution as a marker of portal hypertension.

In portal hypertension more relevant than absolute portal vein diameter may be the absence of normal calibre variations with respiration [45] with a reported sensitivity of up to 0.79. It is clear, however, that there is too much variance in absolute portal vein diameter to be used in the diagnosis of portal venous hypertension. Further, in our experience ultrasound access and compliance with breathing directions are often too poor to obtain reliable and reproducible measurements of portal vein diameter change.

### Doppler ultrasound

Doppler ultrasound can measure hepatic blood flow. In the past, several investigations of the utility of Doppler ultrasound as a noninvasive method of assessing the degree of hepatic fibrosis have been made. The theoretical basis is haemodynamic change in the liver during progression from hepatitis to fibrosis and cirrhosis. Reproducibility of Doppler-derived indices, however, has been poor and correlation between indices and disease stage uncertain. In a well-stratified cohort of 65 patients with biopsy-proven HCV–related liver disease, various hepatic vascular indices were investigated prospectively including the hepatic artery velocity and hepatic artery resistive index, portal vein velocity, portal vein diameter and circumference, portal vein congestive index and hepatic artery–portal vein velocity ratio [46]. In less than half of the patients in this study, reproducible and accurate hepatic artery traces and derived indices could be obtained, and in less than one third of patients an accurate portal vein circumference could be determined. The portal vein velocity could be measured in 62 patients but mean values were near identical for different degrees of hepatitis and cirrhosis. Overall there were no significant differences observed in the Doppler indices with increasing severity of liver disease. The authors conclude that Doppler-derived indices are difficult to reproduce reliably and are therefore of limited clinical value in the assessment of hepatic fibrosis or inflammation.

Hepatic vein waveforms have been used to predict cirrhosis with a tendency for the waveform to be biphasic or monophasic in cirrhosis compared with triphasic in normals. Recently, hepatic vein waveforms were re-evaluated in a series of 120 patients with cirrhosis with a broad range of causes. Flat waveforms occurred in only 3 % of cases; otherwise waveforms were bi- and triphasic. There was no correlation between liver dysfunction and the pattern of hepatic vein waveforms [47]. Variability in venous waveforms is commonly found in clinical practice.

**Fig. 7:**
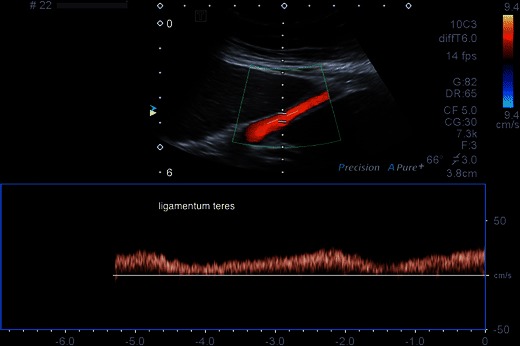
Recanalisation of the paraumbilical vein as shown by venous Doppler signal in the ligamentum teres. This is sensitive and specific for the presence of portal venous hypertension

The main role of Doppler ultrasound is the assessment of portal venous hypertension as a complication of cirrhosis. Doppler ultrasound of the ligamentum teres (Fig. 7) showing hepatofugal venous signal (i.e. a patent paraumbilical vein) and hepatofugal flow in the portal vein are both specific signs and have a high positive predictive value for the presence of portal hypertension [48, 49].

Portal vein velocity as a marker of portal hypertension was assessed in a study of 118 patients and found to be highly variable, ranging between 7 and 83 cm/s. Only 2.6 % of patients in this series had velocities ≤10 cm/s (a threshold suggested by Zoli [50]), and 6.1 % had velocities ≤11 cm/s [49]. Low portal vein velocity is therefore not a very useful sign of portal hypertension. The ratio of cross-sectional portal vein diameter and portal vein velocity, termed the ‘congestion index’ [51], has been proposed as a marker of portal hypertension based on the tendency for portal vein diameter to increase and velocity to decrease. As an index it is not widely used partly because measurement of both parameters of the index is highly variable [49].

Portal venous flow pattern assessment is also valuable in the diagnosis of portal hypertension. Normal portal venous flow is continuous and hepatopetal on Doppler ultrasound with minimal variations due to the cardiac cycle and respiration. Reversed (hepatofugal) portal venous blood flow can be present when the intrahepatic resistance is greater than the resistance of portosystemic collaterals. Continuous hepatofugal flow is present in 8.3 % of patients with cirrhosis [52] and associated with portosystemic shunts [53].

The significance of left gastric vein diameter is unclear. While some correlation with variceal bleeding has been found [54], others found that dilation of the left gastric vein is not necessary for variceal haemorrhage to occur [55].

In a more recent study, a left gastric vein diameter of more than 6 mm was found in 58 % of patients with recent variceal bleed and 12 % of patients without a recent variceal bleed; however this difference was not statistically significant [56].

### Contrast-enhanced ultrasound

Contrast-enhanced ultrasound (CEUS) uses microbubbles as kinetic tracers. CEUS agents have shown to have a good safety profile with a low incidence of side effects. The safety of SonoVue® (Bracco S.p.A., Milan, Italy) has been shown in a retrospective study of 23,188 where only two serious adverse events and no deaths occurred [57]. They are either blood pool agents or combined blood pool and Kupffer phase agents. Different agents have differing properties and they cannot be used interchangeably.

#### CEUS in diffuse liver disease

By measuring vascular transit times and parenchymal enhancement, the severity of liver disease can be assessed. In patients with cirrhosis, the transit times of microbubbles are shortened [58–60]. In addition, it has been shown that using hepatic vein transit times (HVTT) mild hepatitis can be differentiated from moderate and severe hepatitis as well as cirrhosis in HCV-related liver disease [61]. As HVTT shortens with more disease, this method has been suggested as a marker of response to antiviral treatment in HCV [62]. Different agents behave differently, e.g. hepatic vein transit time has been shown to be significantly shorter with SonoVue than with the then available blood pool agent Levovist® (Schering AG, Berlin, Germany) [63]. While the diagnostic accuracy of transit times appears good, the technique is complex and exacting with questionable reproducibility, and the technique is not in mainstream use.

It has been shown that the behaviour of intrahepatic microbubbles depends on the severity of hepatic fibrosis. Sonazoid™ (GE Healthcare, Oslo, Norway), using perfluorobutane as its gas core, is captured in reticuloendothelial tissue. Using this agent in a prospective study of 202 subjects, intrahepatic accumulated microbubbles were used to predict the grade of liver fibrosis [64]. There was significant correlation between the intensity difference and the fibrosis grade. The sensitivity, specificity and efficiency of the intensity difference were 0.88, 0.72 and 0.81 for marked fibrosis, 0.85, 0.91 and 0.89 for advanced fibrosis and 0.97, 0.9 and 0.91 for cirrhosis respectively. Until recently, the CEUS agent Sonazoid™ was only available in Japan.

#### CEUS for lesion characterisation in the setting of chronic liver disease

CEUS can be used in the diagnosis and management of hepatocellular carcinoma, an end point of cirrhosis. The typical features of arterial hyperenhancement and washout as seen on CT or MR can be shown in real time during CEUS. A new concept is defect reperfusion imaging [65] where a Kupffer phase agent such as Sonazoid™ is injected first. This facilitates lesion detection as pathology deficient of Kupffer cells will stand out as an echopoor defect. With a second injection, these defects can then be assessed for arterial enhancement.

### Elastography

There is a correlation between hepatic parenchymal pathology and liver stiffness. As a surrogate marker of fibrosis and cirrhosis, the measurement of liver stiffness forms the basis of elastography. Stiffness, or the rigidity of an object, is the extent to which it resists deformation in response to a force applied. Elasticity is the tendency of solid materials to return to their original shape after being deformed by a force applied and removed. In elastography, such force is coupled with a system that measures the deformities caused by the force. Ultrasound elastography techniques include transient elastography (FibroScan®), acoustic radiation force impulse imaging (ARFI), shear wave mode elastography and strain elastography. A recent review by Frulio and Trillaud provides details on different elastography techniques as well as a brief discussion on the main serum markers to assess for fibrosis [66]. A 2013 review by the Japan Society of Ultrasonics in Medicine discussed fundamental principles of elastography methods and physics of tissue elastic properties [67].

#### Transient elastography

**Fig. 8:**
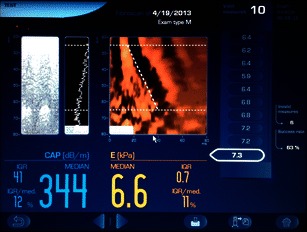
User interface of Fibroscan. Example of liver stiffness measurement: mean 6.6 kPa falls in the F0 or F1 category (absent or mild fibrosis)

This ultrasound method is based on Hooke’s law, which states that the force required to compress or extend a spring is proportional to the distance compressed or extended. This law can be applied to a variety of materials including liver tissue. Transient elastography (TE) is the technique used with the FibroScan® ultrasound unit (Echosens S.A.S.U., Paris/France). In practice, FibroScan® uses a 5 MHz ultrasound transducer mounted on the axis of a vibrator, placed in a right intercostal space with the patient lying supine and the right arm in maximal abduction. A 50-Hz vibration with an amplitude of 2 mm created propagates into the liver as elastic shear waves. The speed of wave propagation is proportional to the tissue stiffness and is measured by pulse-echo ultrasound. This measured speed is then converted to the Young modulus using a simplified equation and expressed in kiloPascals (kPa) [68]. Ten successful measurements are to be obtained in any given patient and the median value in kPa is calculated (Fig. 8). The stiffer the tissue is, the higher the speed of wave progression.

There are a number of limitations in TE. The major disadvantage in comparison with other elastography techniques is that no ultrasonographic visualisation of the location of the measurement is possible. It is impossible to perform in patients with ascites. The liver stiffness measurements may be influenced by acute liver injury [69–71] indicated by acute aminotransferase flares, extrahepatic cholestasis [72], central venous pressure [73], beta-blockers [74] and food intake [75]. TE is difficult to perform in obese patients or those with a narrow intercostal space. In addition, it is operator dependant. In an analysis of 13,369 cases, a 15.8 % rate of unreliable and 3.1 % rate of failed TE measurements were reported [76]. This corresponds to liver stiffness measurements using TE being uninterpretable in nearly one in five cases. The main reasons were found to be obesity and limited operator experience. In addition, patient age >52 years and presence of type 2 diabetes mellitus were independently associated with measurement failure and female gender and arterial hypertension were independently associated with unreliable LS measurements. In order to mitigate the high rate of unreliable measurements in obese patients, a lower frequency (2.5 MHz) XL probe has been developed that has achieved reliable measurements in 61 % of obese patients in whom measurements were unreliable using the conventional M probe [77]. The use of an XL probe in obese patients increased the rate of reliable measurements from less than 50 % (M probe) to approximately 75 %. Similarly for patients with smaller build and children, an S probe with a higher frequency and shallower sampling depth is being developed [78].

The diagnostic performance of TE has been assessed for a variety of aetiologies: HCV [79], HIV/HCV [80, 81], HBV [82], NAFLD [83] and alcoholic liver disease [84]. In a meta-analysis including 40 studies an overall sensitivity and specificity of 0.79 and 0.78 for *F*=2 stage and 0.83 and 0.89 for cirrhosis were reported [85].

In a meta-analysis comprising 18 studies including 2,772 patients exclusively with HBV, the mean area under the ROC curve for the diagnosis of *F*=2 was 0.859, for *F*=3 0.887 and for *F*=4 0.929. The estimated cutoff for *F*=2 was 7.9 (range, 6.1–11.8) kPa, with a sensitivity of 74.3 % and specificity of 78.3 %. For *F*=3, the cutoff value was determined to be 8.8 (range, 8.1–9.7) kPa, with a sensitivity of 74.0 % and specificity of 63.8 %. The cutoff value for *F*=4 was 11.7 (range, 7.3–17.5) kPa, with a sensitivity of 84.6 % and specificity of 81.5 % [86].

The optimal cutoff values for advanced fibrosis and cirrhosis differ according to aetiology and are subject to debate as even within one aetiology a broad range of cutoff values exist.

In addition to using TE in the assessment of fibrosis and cirrhosis, a new method has emerged for the quantification of steatosis. The amplitude of ultrasound waves decreases as they propagate through the liver. This attenuation is measured using the Fibroscan M probe and termed the controlled attenuation parameter (CAP™). Initially assessed in a pilot study of 115 patients, CAP™ detected >10 % (S1), >33 % (S2) and >67 % (S3) steatosis with areas under the ROC curves of 0.91, 0.95 and 0.89 respectively. The presence of fibrosis did not affect the CAP™ values [87]. Similar performances were found in further studies, e.g. with areas under the ROC curves of 0.84, 0.86 and 0.93 for >10 % (S1), >33 % (S2) and >67 % (S3) steatosis respectively [88] or with areas under the ROC curves of 0.79, 0.76 and 0.70 for >5 %, >33 % and >67 % steatosis respectively [89]. There is evidence that the aetiology of steatosis does not affect the accuracy of CAP™ [90].

The grades of steatosis used in the above studies correspond to the original proposal for grading by Brant et al. [5] with the exception of S1 where the degree of steatosis is <33 %.

Early studies of CAP™ have shown promising results for quantification of hepatic steatosis, although some have reported limited accuracy in the range of severe disease and high failure rates. In a large prospective study of 5323 CAP™ examinations the overall failure rate was 7.7 %, and 33 % in elderly females with diabetes and hypertension [91]. Further studies are required for validation and refinement of its use.

In summary, TE is the longest established and most validated technology. It has excellent diagnostic accuracy for cirrhosis and good accuracy for the detection of early cirrhosis. It is user friendly and has a high patient acceptance. There are a number of limitations and pitfalls, and dedicated non-imaging ultrasound hardware is required. The main limitations are high BMI and ascites, and there is a variable but not insignificant failure rate.

#### Acoustic force radiation impulse

Acoustic Force Radiation Impulse (ARFI) technology is a technique that has been incorporated into an imaging ultrasound unit. A 5 mm × 10 mm region of interest (ROI) cursor is placed during real-time B-mode scanning. The technique was termed shear wave elastography at a point (pSWE) given the small size of the ROI cursor [68].

**Fig. 9:**
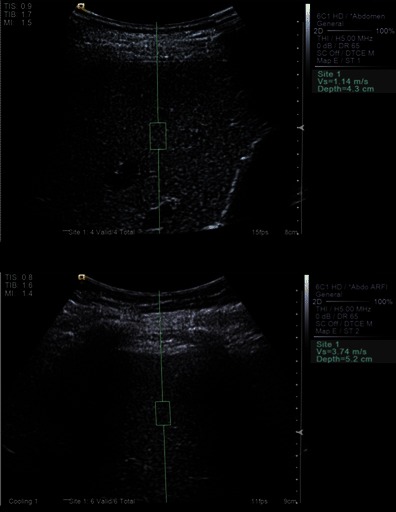
Elastography (ARFI). Example of normal liver with ARFI value Vs = 1.14 m/s indicating absent or mild fibrosis (F0 or F1), and example of abnormal liver with ARFI value Vs = 3.74 m/s indicating F4 = cirrhosis

The tissue in the ROI is excited with a short duration (262 µs) fixed frequency (2.67 MHz) ultrasound pulse to displace tissue locally. The resultant shear wave propagates laterally with a velocity that is proportional to the square root of tissue elasticity and detected with ultrasound-based correlation methods. The speed of the shear wave is measured directly in meters per second and is displayed on the screen (Figs. 9). Unlike TE, ARFI is not impeded by mild to moderate perihepatic ascites. As the technology is implemented in an ultrasound machine, it can be part of any B-mode liver ultrasound scan without switch of equipment (e.g. Virtual Touch™ Tissue quantification in Siemens ACUSON S2000, Siemens Medical Solution, Erlangen, Germany; Elasto Q Philips iU22, Philips, Best, The Netherlands). In addition, the site of sampling is selectable and known precisely. In practice, ARFI is often carried out as part of liver ultrasound. Segments V/VI or VII/VIII are most suited for sampling, with the left liver lobe best avoided [68, 92, 93]. The two main reasons for better suitability of the right liver for ARFI sampling are the disruption of shear waves by excessive tissue motion secondary to cardiac pulsation affecting the left lobe, and readings in segments II/III are likely to be taken from tissue closer to the capsule, an area known to be more fibrous than deeper zones.

The success rate (SR) has been measured by the ratio of successful acquisitions over the total number of acquisitions and interquartile range (IQR) interval, defined as the difference between the 75th and the 25th percentile. These are quality technical parameters that have not been recommended by the manufacturer (Siemens); however it has been shown that these values (IQR <30 % of the median velocity and a SR ≥60 %) can be used to improve the value of ARFI [94].

Significant inter-observer correlation between ARFI measurements with *r* = 0.874 has been shown [93]. In addition, in a later study the intra-operator (intra-class correlation coefficient ICC = 0.9) and inter-operator (ICC = 0.81) reproducibility of ARFI was shown to be good [95] in a study consisting of patients with cirrhosis and fibrosis of various aetiologies and healthy volunteers alike.

ARFI was first used and validated in patients with chronic HCV. In an early pilot study with 86 patients with HCV, the area under the ROC curve for the diagnosis of *F* ≥ 2 was 0.82 and for the diagnosis of *F* = 4 was 0.91 [96]. In a study of 274 patients with HCV areas under the ROC curves to predict *F* ≥ 2, *F* ≥ 3 and *F* = 4 were 0.893, 0.908 and 0.937 respectively [97]. A multicentre study from 2012 confirms ARFI as a good method for predicting cirrhosis in HCV patients [98]; however a problem area remains with stages F0-F1 where overlap of measurements persists.

In a pooled meta-analysis assessing the performance of ARFI in the staging of liver fibrosis due to HCV, HBV and NASH, patient data were available from eight studies including 518 patients, all with biopsy as a reference method. The mean diagnostic accuracy of ARFI expressed as area under the ROC curve was 0.87 for the diagnosis of significant fibrosis (*F* ≥ 2), 0.91 for the diagnosis of severe fibrosis (*F* ≥ 3) and 0.93 for the diagnosis of cirrhosis [99]. However there have been differences in the areas under the ROC curves for HCV vs. HBV with 0.88 vs. 0.79 for *F* ≥ 2, 0.90 vs. 0.83 for *F* ≥ 3 and 0.92 vs. 0.90 for *F* = 4, respectively. In a further meta-analysis addressing the efficiency of ARFI for the staging of liver fibrosis, 36 studies including 3,951 patients were included. The mean diagnostic accuracy of ARFI expressed as area under the ROC curve was 0.84 for the diagnosis of significant fibrosis (*F* ≥ 2), 0.89 for the diagnosis of severe fibrosis (*F* ≥ 3) and 0.91 for the diagnosis of liver cirrhosis (*F* = 4) [100].

A number of studies have compared the diagnostic performance of ARFI with TE. In a cohort of 139 consecutive patients with chronic HCV, ARFI has been shown to be more accurate than TE for both significant and more severe stages of fibrosis [101]. In prior studies, for the prediction of severe fibrosis, ARFI and TE appear to have the same diagnostic performance [96, 102, 103]. In the prediction of *F* ≥ 1 or *F* ≥ 2, however, TE outperformed ARFI in two studies [102, 103], while ARFI and TE were equal for all F stages [96]. In a recent prospective study of 321 consecutive patients undergoing assessment with ARFI, TE with the Fibroscan M- and XL-probes, and liver biopsy within 1 month after elastography, ARFI was shown to be reliable in the assessment of liver fibrosis, especially in nonobese patients [104]. In this study, ARFI had a measurement failure rate of 0 %, compared to 2.3 % and 11.2 % for the XL and M probes of Fibroscan respectively. The M probe slightly outperformed ARFI in the diagnosis of moderate fibrosis with areas under the ROC curves of 0.81 and 0.88.

Data on the influence of different confounding factors on ARFI measurements are limited. Liver stiffness is affected by both physiological and pathological processes. Like TE, ARFI values are influenced by acute inflammation [105, 106]. While TE is affected by both moderate and high aminotransferase levels, ARFI appears to be affected by high levels as shown in a large multicentre study [106]. It has been shown that food intake significantly increases ARFI values [107] and measurements should therefore be performed in a fasting state. While initially validated for HCV, there is growing evidence that ARFI is accurate in predicting NAFLD fibrosis. Significant correlation between ARFI values and the degree of fibrosis has been shown [108, 109]. More recently, ARFI has been investigated as a discriminating method between NASH and simple steatosis [110, 111]. Significantly higher ARFI velocities in NASH, and areas under the ROC curves of 0.87–0.9 for the differentiation between NASH and simple steatosis were found.

In conclusion, ARFI is easy to perform with good reproducibility. The technology is incorporated in conventional ultrasound systems, and results are available within seconds. This method has been shown to have good diagnostic accuracy for the staging of fibrosis grades *F* ≥ 2 and *F* ≥ 3 and excellent diagnostic accuracy for *F* = 4. Drawbacks are a small pre-determined measurement area, and validation is not as extensive as for TE as yet.

#### Two-dimensional shear wave elastography

**Fig. 10:**
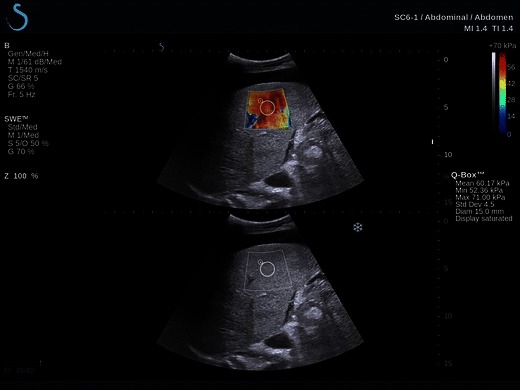
Example of grade F3 fibrosis as shown on shear wave elastography (SWE), a quasi real-time two-dimensional map of tissue elasticity superimposed in colour on B-mode images

The shear wave elastography (SWE) Aixplorer™ ultrasound system (SuperSonic Imagine S.A, Aix-en-Provence, France) generates shear waves in tissue from the acoustic radiation force obtained with focussed ultrasound pulses. Hard- and software are proprietary [68]. Serial pulses create plane shear waves that propagate transversely. Plane wave imaging is used to determine the speed of shear waves. Tissue elasticity is related to shear wave velocity and expressed in kPa. It allows the generation of a quasi real-time two-dimensional map of tissue elasticity, which is superimposed in colour on B-mode images at a low frame rate of approximately 1/s. As in most SE systems, the range of colours is from red (soft tissue) to blue (hard tissue). On a frozen image, mean and standard deviation of tissue stiffness can be displayed within a set ROI (Fig. 10). A potential advantage of 2D-SWE real-time information on shear wave speed is the display and measurement in a two-dimensional area as opposed to a point or line fashion as ARFI or TE does. Only a few studies have been reported to date. In a study limited to 121 patients with HCV, SWE was superior to TE in the detection of *F* > 2 [112]. In a further study limited to 113 patients with HCV, SWE achieved areas under the ROC curves of 0.95, 0.96 and 0.97 for the prediction of *F* ≥ 2, *F* ≥ 3 and *F* = 4, respectively, comparing favourably to TE where areas under the ROC curves of 0.85, 0.86 and 0.94 were achieved for the prediction of *F* ≥ 2, *F* ≥ 3 and *F* = 4, respectively [113]. In a study comparing the performance of SWE to that of TE (M and XL probes), the applicability of SWE in patients with ascites was 86 % (55 % for TE) [114]. Although these initial results are promising, more studies are necessary to validate the technique for other aetiologies, in other centres and for other factors that may influence measurements such as steatosis and inflammation.

#### Strain elastography (real-time tissue elastography)

Strain elastography is technically distinct from transient elastography, using a conventional ultrasound transducer to collect signals with and without distortion of tissue. This distortion is achieved by way of external compression, which may be applied free hand, with the US transducer, or by endogenous movements (arterial pulsation or cardiac motion). While initially used for the assessment of focal lesions in the pancreas, prostate, breast and thyroid, this method is now also being used for liver fibrosis.

B-mode ultrasound is used to select the area of liver to the sampled. A colour map is then laid over the B-mode image in real time—hence the term real-time elastography, representing areas of different tissue deformabilities, which in turn are related to stiffness. Strain elastography is designed to provide a qualitative measurement of stiffness. In most systems the display conventionally ranges from red (soft tissue) to blue (hard tissue). SE is the only elastographic technique that works in real time at up to 20 frames/s and on a large field of view. Attempts at semi-quantitative interpretation have been made using scoring systems, e.g. by encoding the colour map and assigning values, e.g. between blue = 0 and red = 255 [115], or histogram analysis [116]. Another approach is the use of a specifically designed software for quantification of the colour map output [117], achieving area under the ROC curve values of 0.75 for the diagnosis of *F* ≥ 2, 0.73 for the diagnosis of severe *F* ≥ 3 and 0.69 for the diagnosis of cirrhosis. Studies have predominantly involved patients with chronic HCV [115, 117–120], but also NASH fibrosis and cirrhosis [121]. The optimal approach of quantifying strain elastography, as well as standardisation of the stressor modality are yet to be determined. A recommendation for clinical use in the liver awaits more evidence and validation.

## Conclusion

Conventional B-mode ultrasound remains a valuable tool in first-line screening of patients with chronic liver disease, supplemented by portal venous Doppler studies. While elastographic methods of TE and ARFI have now been validated for the assessment of fibrosis, other elastographic technologies are emerging and are likely to become more widely applied.

## Future perspective

The increasing prevalence of chronic liver disease, including fatty liver disease, together with the need to minimise invasive liver biopsies will continue to provide substantial drive for ultrasound research and development.

- *Conventional B-mode ultrasound*
  - Quantification of echointensity in the diagnosis of fatty liver disease is an area of recent interest. Standardisation of technique and interobserver reproducibility will need to be addressed.
  - Quantitative and semi-quantitative assessment of echotexture in the diagnosis of fibrosis and fatty liver disease is being developed
- *Doppler ultrasound*
  - The role of Doppler ultrasound in chronic liver disease is likely to remain principally in the diagnosis of portal venous hypertension
- *Contrast-enhanced ultrasound*
  - Kupffer-phase contrast agents can be used in the grading of fibrosis as well as in the detection of focal lesions, and it is hoped that these agents will become more widely available.
- *Elastography*
  - More widespread use of the two most extensively validated and established techniques of TE and ARFI is expected, and further shear wave elastography techniques are likely to become more widely adopted and evaluated
